# Fighting Back Against Childhood Obesity Through the Cape May County Children’s Health Summit

**Published:** 2004-09-15

**Authors:** Marilou Rochford, Elizabeth Kaminsky

**Affiliations:** Family and Consumer Sciences Educator, Rutgers Cooperative Extension of Cape May County, Associate Professor, Rutgers, The State University; Family and Consumer Sciences, Rutgers Cooperative Extension of Cape May County, Cape May Court House, NJ

## Background

Obesity is a fast-growing health issue affecting children and adolescents across the nation. Rutgers Cooperative Extension (RCE), along with agency and community partners, implemented a collaborative solution aimed at raising awareness, providing education, and effecting workable strategies for preventing obesity in children and families in the Cape May County, New Jersey, community.

Because we regularly work with children and families, we knew that our clientele mirrored the national statistics on obesity, which state that 13% of children aged six to 11 years and 14% of adolescents aged 12 to 19 in the United States are overweight (1). These numbers represent a nearly three-fold increase for adolescents in the past two decades, the effects of which can be devastating both physically and emotionally. Adult health issues, such as high cholesterol, high blood pressure, and type 2 diabetes, have increased dramatically in children (2). Further troubling statistics show that overweight adolescents have a 70% chance of becoming overweight or obese adults, the likelihood of which increases to 80% if at least one parent is overweight or obese (2). Overweight children suffer emotionally, too, facing social discrimination, poor self-esteem, and depression (3).

In fulfilling our mission as an educational outreach organization, RCE responded to community needs for research-based information on the causes, treatments, and prevention of childhood obesity. RCE organized a comprehensive educational forum, bringing together resources from Rutgers University, the New Jersey State Department of Education, New Jersey State Department of Human Services, United States Department of Agriculture, New Jersey Department of Agriculture, and many other local and regional entities.

## Context

Cape May County (CMC) is situated at the southernmost tip of New Jersey. It is home to roughly 100,000 year-round residents, and the summer tourist population swells to almost 700,000. It is a unique geographic mix of seashore resorts and rural farmland. The county's location and tourism-driven economy afford little opportunity for adult residents to obtain continuing education. Access to public transportation is limited, and the year-round population is stratified across many smaller towns. There is no single urban center, and there are no four-year colleges. Additionally, the tourism-driven economy causes periods of both high employment and high unemployment, depending upon the season; the overall unemployment rate in 2000 was 8.2% (4). The county has only one hospital and one primary mental-health counseling provider.

More than 22% of the county's total population is made up of children under 18 years old, and 20% of the total population is aged 65 and older, much higher than the state average. Almost 9% of adults live below the poverty level, with an additional 12% of related children also living below poverty. In each of these cases, CMC's poverty numbers are slightly higher than the state average (4). Additionally, skyrocketing real-estate prices have created a significant shortage of affordable housing for mid- to low-income families and individuals. The uncommon blend of residents and their specialized needs creates challenges for the social workers, school personnel, health care professionals, child care providers, nutrition educators, counselors, family therapists, and other professionals or volunteers working with CMC youth and families. In our interactions with all these individuals and groups, the discussion has consistently focused on seeking the best ways we can all help our county's children and families who face bullying, discrimination, and physical health problems as a result of being overweight.

## Methods

**Figure:**
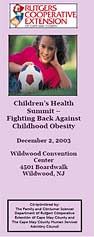
The registration brochure from the Children's Health Summit Outside of Brochure Schedule 8:30 AM – Registration, continental breakfast and exhibits 9:00 AM – Introduction and opening remarks: The causes and consequences of obesity in children 9:30 AM – Staying healthy in a supersized world - Strategies for successful weight management 10:15 AM – Helping kids cope – Finding workable family solutions for addressing the emotional issues of overweight children 11:00 AM – Health and student achievement – Understanding the mind/body connection for children 12:00 Noon – Lunch, local resource sharing and exhibits 1:00 PM – Active kids/healthy kids – Exploring the physical, cognitive and emotional benefits of exercise 2:00 PM – Evaluations and wrap up In partnership with:
Cape May County Family and Consumer Sciences Advisory BoardNew Jersey Department of EducationCape Counseling Services, IncAmerican Association of Family and Consumer SciencesNational Extension Association of Family and Consumer Sciences, New Jersey AffiliateNJ Department of AgricultureCape May County Board of AgricultureNJ Nutrition CouncilNJ Five-A-DayNew Jersey Obesity Group Family and Consumer Sciences Department Rutgers Cooperative Extension of Cape May County 4 Moore Road Cape May Court House, NJ 08210 Phone: 609-465-5115, ext. 609 or 611 Fax: 609-465-5953 E-mail: rochford@aesop.rutgers.edu Logo: State University of New Jersey Rutgers Logo: Rutgers Cooperative Extension of Cape May County Image: Smiling girl on the lawn with a soccer ball Children’s Health Summit – Fighting Back Against Childhood Obesity December 2, 2003 Wildwood Convention Center 4501 Boardwalk Wildwood, NJ Co-sponsored by: The Family and Consumer Sciences Department of Rutgers Cooperative Extension of Cape May County and The Cape May County Human Services Advisory Council Inside of Brochure Helping build healthy kids, inside and out Image: Smiling mom and daughter Today’s children face a variety of challenges in their lives. Problems like diabetes, high blood pressure and high cholesterol used to be limited to adults. Now, those health issues are quickly becoming realities for children. Overweight kids suffer emotionally as well, facing increased risks of suicide, depression, bullying and low self-esteem. To address these issues and present solutions for preventing and treating childhood obesity, the Family and Consumer Sciences Department of Rutgers Cooperative Extension, in partnership with Cape May County Human Services Advisory Council, have organized this Children’s Health Summit. Noted speakers from Rutgers University and the New Jersey Department of Education will discuss the issues, present the latest research and offer solutions for parents, teachers, coaches, health care providers, counselors, and all caring adults who work with children. Local professionals will be on hand to discuss community resources. Continental breakfast and light lunch will be provided. Continuing education credits will be available. Image: Smiling boy on the lawn with a book Registration Form Children’s Health Summit *Fighting Back Against* *Childhood Obesity* Registration deadline Nov. 18th Name ____________________________ Address ____________________________ Phone ____________________________ E-mail address ____________________________ There is no charge to attend. □ I am interested in obtaining continuing education credit* □ I am NOT interested in obtaining continuing education credit Return this form to: Family and Consumer Sciences Department Rutgers Cooperative Extension of Cape May County 4 Moore Road Phone: 609-465-5115, ext. 609 or 611 Fax: 609-465-5953 Email: rochford@aesop.rutgers.edu

RCE faced certain constraints in producing a comprehensive educational program that would raise public awareness and build skills for dealing with the problem of childhood obesity. There was no money within our own operating budget to fund a conference complete with multiple workshops, exhibitors, food service, and advertising. We secured a grant from the county's Human Services Advisory Council and received administrative support from Cape Counseling Services, Inc. Through a combination of coalition building, grant solicitation, and enormous volunteer effort, we were able to host a full day of training for more than 90 professionals, parents, teachers, school nurses, counselors, dietitians, family and consumer sciences educators, and other interested adults. Very few community educational events draw more than 40 or 50 participants at a time, making our event significant in its scope and outreach.

We considered many factors in coordinating this event. We selected midweek in early December, traditionally a quiet time for meeting facilities in our area. For many residents, seasonal employment is winding down, leaving time available for training. Schools are in session, so parents with school-age children have less need for child care. Professionals were able to receive continuing education credits, which served as a two-fold benefit: people with year-end continuing education deadlines were able to fulfill those requirements without having to travel out of the area. (Very limited accredited training is offered in this geographic area.)

The newly opened Wildwood Convention Center provided a central location with plenty of space and a state-of-the-art facility. The site was appropriate for a number of reasons. There are very few locations in our county that can host more than 50 people. Wildwood is one of our neediest communities. Residents move around easily within the town, but have difficulty traveling any distance outside the community. The off-season time frame made the location affordable and accessible. Parking was plentiful and traffic was nonexistent, unlike in summer months when both factors generate concern. Because the conference dealt with battling obesity, a healthy menu for breakfast and lunch had to be developed and produced within a very limited budget. The convention center staff was committed to the conference mission and worked closely with us to provide healthful, cost-effective meals.

The educational components of the day were carefully selected to bring the most current research-based information to participants from a variety of backgrounds, interests, and abilities. Elected officials from the state assembly and the county Board of Chosen Freeholders welcomed participants and added their support and endorsement. Speakers were drawn from Rutgers University and the New Jersey Department of Education, based on their experience with nutrition, health, and child development. Workshops covered such topics as the pitfalls of our supersized society, the emotional impact of obesity, and the link between health and student academic achievement. Local organizations, such as the Middle Township Schools, were called upon to showcase their efforts toward combating obesity. This school system's innovative fitness program for elementary school children earned it a $250,000 federal grant. The same school system also works with adolescents, promoting *Girls on the Run*, a noncompetitive activity program for young women, a group in need of support and encouragement for healthy self-image. Local resource professionals from Burdette Tomlin Memorial Hospital, Cape Counseling, Inc, Cape May County School Nurses' Association, Cape Fitness, and Lower Township Elementary School Food Services spotlighted their organizations to educate participants about programs and services available. The RCE Department of Family & Consumer Sciences and 4-H Youth Development provided research-based publications and information from national, state, and local sources. Representatives from the New Jersey Department of Agriculture, the New Jersey Department of Education, and the New Jersey Department of Health and Senior Services exhibited information and resources from the state level.

## Consequences

The event generated local and regional press coverage, including a feature interview and a nightly news story on our area's NBC affiliate. Newspaper and television coverage helped to heighten public awareness and extend the message about childhood obesity to a broader audience.

Post-conference evaluations assessed participants' attitudes, knowledge, and potential for behavior change. Participants shared their commitment to becoming better role models for children. Suggestions for setting good examples included watching less television, spending less time on the computer, being more active, and making healthier food choices such as drinking milk and eating whole grains.

Participants increased their knowledge about the physical and emotional effects of obesity in children. Areas highlighted included understanding the health risks of obesity and addressing children's emotional needs for acceptance, support, and encouragement. We challenged participants to think about what they would do to become an active part of the solution. Participants were called upon to commit to one or more of the following behaviors: eat a healthy breakfast, help children find ways other than food to handle setbacks or successes, play and be physically active with children, select standard rather than supersized food portions, teach children to accept all body shapes and sizes, urge clubs and organizations to sell healthy foods or nonfood items for fundraising events, set a good example by eating a balanced diet, avoid using exercise as a punishment, let children know that they are loved unconditionally, and recognize children for their positive qualities, strengths, and abilities rather than for their physical appearance.

Other suggested solutions involved taking a family approach, that is, encouraging parents to involve all family members in decisions about diet and exercise rather than singling out family members who may be overweight. Participants discussed giving parents the tools to focus on healthy lifestyles and to prevent obesity problems before they begin. Ideas included encouraging family mealtimes and physical activities and discouraging a focus on media stereotypes for body image role models.

Community-wide solutions were also discussed. Serving better food in schools, limiting snacks or changing snack choices, and questioning the need for vending machines in schools were just a few of the many suggestions of how individuals and organizations could respond. To maintain the momentum for further activities on this topic, participants were asked about their willingness to stay involved and work with RCE as a member of our Building Healthy Kids Coalition. More than 65% of participants indicated their willingness to stay involved.

## Interpretation

Over the last several years, RCE has positioned itself as a leader in identifying important societal trends and addressing the needs of CMC families. Our reputation for effective collaborations and high-quality programs has helped us maintain lasting partnerships that benefit all of our clientele.

The true value in our family-centered, community-focused approach lies in the strong connections we are able to forge with the people of our county. Helping children lead healthier lives is a topic everyone can feel good about. Our grass-roots, cooperative efforts allowed many diverse organizations to play a role in the conference. Legislators, county officials, local media, schools, and faith-based organizations are supportive of future efforts to reduce and prevent childhood obesity.

The Building Healthy Kids Coalition has met monthly since the conference. The overall goal of the coalition is to strengthen families in a variety of ways that will encourage and support the development of healthy children, including their physical, emotional, and environmental health. Under RCE's leadership, the coalition plans to develop and implement a countywide walking program to promote physical activity for CMC youth and adults during 2004–2005. The walking program was chosen for numerous reasons, including that it will be easy to implement and can be undertaken at minimal cost. In addition, walking costs nothing for participants to continue after the formal program ends, it can be a lifelong activity, it is an activity that sets a good example for children, and it is something that everyone can do. Future projects will be undertaken based on community need.

RCE will continue its efforts to bring research-based education and information to families dealing with obesity issues. Through collaboration with its many partners, RCE will raise awareness and provide workable solutions for the prevention and treatment of childhood obesity.

## References

[1] Centers for Disease Control and Prevention Prevalence of overweight among children and adolescents – United States 1999-2000 [Internet].

[2] U.S. Department of Health and Human Services (2001). The Surgeon General's call to action to prevent and decrease overweight and obesity – United States 2001.

[3] Schwimmer JB, Burwinkle TM, Varni JW (2003). Health-related quality of life of severely obese children and adolescents. JAMA.

[4] Cape May County Planning Department (2003). Cape May County data book.

