# Supplementary material for: Fighting Back Against Childhood Obesity Through the Cape May County Children’s Health Summit
Source: Prev Chronic Dis. 2004 Sep 15;1(4):A17. (PMC1277957)
Supplement: Supplementary file 1 [file 04_0067_01.pdf]

Schedule

8:30 AM – Registration, continental breakfast and exhibits

9:00 AM – Introduction and opening remarks: The causes and consequences of obesity in children

9:30 AM – Staying healthy in a super-sized world - Strategies for successful weight management

10:15 AM – Helping kids cope – Finding workable family solutions for addressing the emotional issues of overweight children

11:00 AM – Health and student achievement – Understanding the mind/body connection for children

12:00 Noon – Lunch, local resource sharing and exhibits

1:00 PM – Active kids/healthy kids – Exploring the physical, cognitive and emotional benefits of exercise

2:00 PM – Evaluations and wrap up

In partnership with:

- Cape May County Family and Consumer Sciences Advisory Board
- New Jersey Department of Education
- Cape Counseling Services, Inc.
- American Association of Family and Consumer Sciences
- National Extension Association of Family and Consumer Sciences, New Jersey Affiliate
- NJ Department of Agriculture
- Cape May County Board of Agriculture
- NJ Nutrition Council
- NJ Five-A-Day
- New Jersey Obesity Group

Family and Consumer Sciences Department
Rutgers Cooperative Extension of Cape May County
4 Moore Road
Cape May Court House, NJ 08210

Phone: 609-465-5115, ext. 609 or 611
Fax: 609-465-5953
Email: rochford@aesop.rutgers.edu

THE STATE UNIVERSITY OF NEW JERSEY
RUTGERS

RUTGERS COOPERATIVE EXTENSION
N.J. AGRICULTURAL EXPERIMENT STATION
RUTGERS, THE STATE UNIVERSITY OF NEW JERSEY
NEW BRUNSWICK

Distributed in cooperation with U.S. Department of Agriculture in furtherance of the Acts of Congress on May 8 and June 30, 1914. Rutgers Cooperative Extension works in agriculture, family and consumer sciences, and 4-H. Adesoji O. Adelaja, Director of Extension. Rutgers Cooperative Extension provides information and educational servicesto all people without regard to race, color, national origin, gender, religion, age, disability, political beliefs, sexual orientation, or marital or family status (Not all prohibited bases apply to all programs.) Rutgers Cooperative Extension is an Equal Opportunity Program Provider and Employer.

RUTGERS COOPERATIVE EXTENSION
OF CAPE MAY COUNTY

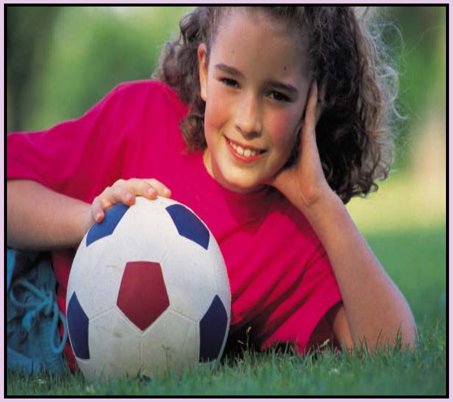

Children's Health Summit – Fighting Back Against Childhood Obesity

December 2, 2003

Wildwood Convention Center
4501 Boardwalk
Wildwood, NJ

Co-sponsored by:
The Family and Consumer Sciences Department of Rutgers Cooperative Extension of Cape May County and The Cape May County Human Services Advisory Council

Helping build healthy kids, inside and out

Today's children face a variety of challenges in their lives. Problems like diabetes, high blood pressure and high cholesterol used to be limited to adults.

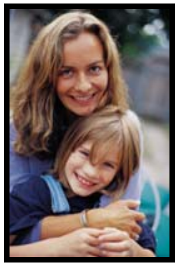

Now, those health issues are quickly becoming realities for children. Overweight kids suffer emotionally as well, facing increased risks of suicide, depression, bullying and low self-esteem.

To address these issues and present solutions for preventing and treating childhood obesity, the Family and Consumer Sciences Department of Rutgers Cooperative Extension, in partnership with Cape May County Human Services Advisory Council, have organized this Children's Health Summit.

Noted speakers from Rutgers University and the New Jersey Department of Education will discuss the issues, present the latest research and offer solutions for parents, teachers, coaches, health care providers, counselors, and all caring adults who work with children. Local professionals will be on hand to discuss community resources.

Continental breakfast and light lunch will be provided. Continuing education credits will be available.

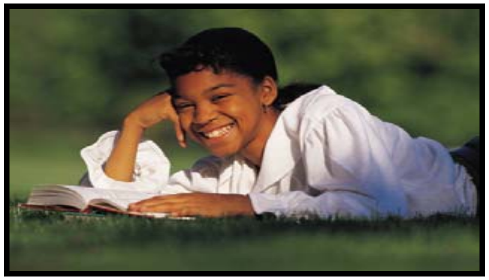

Registration Form
Children's Health Summit
Fighting Back Against Childhood Obesity
Registration deadline Nov. 18th

Name ______________________

Address ______________________

____

____

____

Phone ______________________

E-mail address ______________________

There is no charge to attend.

❑ I am interested in obtaining continuing education credit *
❑ I am NOT interested in obtaining continuing education credit

Return this form to:

Family and Consumer Sciences Department
Rutgers Cooperative Extension of Cape May County
4 Moore Road
Phone: 609-465-5115, ext. 609 or 611
Fax: 609-465-5953
Email: rochford@aesop.rutgers.edu
